# Correlation between the level of cholesteryl ester transfer protein in follicular fluid with fertilization rates in IVF/ ICSI cycles

**Published:** 2011

**Authors:** Amir Mehdizadeh, Ali Rahimipour, Laya Farzadi, Masoud Darabi, Vahideh Shahnazi, Amir-Mansour Vatankhah, Zahra Golmohamadi, Mohammad Nouri

**Affiliations:** 1 Research Center for Pharmaceutical Nanotechnology, Tabriz University of Medical Sciences, Tabriz, Iran.; 2 Laboratory of Chromatography, Department of Biochemistry and clinical Laboratories, Faculty of Medicine, Tabriz University of Medical Sciences, Tabriz, Iran.; 3 Department of Laboratory Medicine, Shahid Beheshti University of Medical Sciences, Tehran, Iran.; 4 Women’s Reproductive Health Research Center, Alzahra Hospital, Tabriz University of Medical Sciences, Tabriz, Iran.; 5 Drug Applied Research Center, Tabriz University of Medical Sciences, Tabriz, Iran.

**Keywords:** *Infertility*, *IVF*/*ICSI*, *Apolipoprotein A*-*I*, *CETP*

## Abstract

**Background:**

Follicular fluid (FF) plays an important role in oocytes and embryo development, which may contribute to IVF/ICSI success rate.

**Objective:**

The aim of this study was to investigate the correlation between cholesteryl ester transfer protein (CETP) level in FF and the success rate of IVF/ICSI.

**Materials and Methods:**

In a cross-sectional study, FF samples, FF samples were obtained from 100 patients referred to Tabriz Alzahra Hospital. Seventy-nine subjects underwent IVF and the remaining 21 underwent ICSI. The levels of high-density lipoprotein cholesterol (HDL-C), apolipoprotein A-I and CETP were measured using enzymatic, turbidometric and ELISA methods respectively.

**Results:**

Analysis of the subgroups with different levels of CETP showed a significant lower level of CETP in the subgroup with the lowest number of mature oocytes (p<0.05). The level of CETP was also considerably lower (18%, p=0.05) in subjects with<50% oocytes fertilization ratio than subjects with >70% of this ratio.

**Conclusion:**

While no association was found for pregnancy, the amount of CETP in FF was associated positively to the maturity and the percentage of oocyte fertilization.

## Introduction

Infertility is a multifactorial disorder with lifestyle and environmental traits playing important role (1). Assisted conception techniques such as IVF/ICSI are used to treat a range of infertilities. However, these techniques are not always successful and potential factors contributing to a successful IVF/ICSI treatment are being investigated (2).

Follicular fluid (FF) which is necessary for the development of the female gamete, may also affect the fertilization and embryonic development. Therefore, FF is occasionally used as a medium supplement in clinical IVF (3). High density lipoprotein (HDL) particles of FF are the most important agent for transferring cholesterol to granulosa cells (4). So, HDL cholesterol (HDL-C) is the most important resource of cholesterol for progesterone synthesis after LH surge (5, 6). Apolipoprotein A-I (apo A-I) is the major apolipoprotein in FF, which is associated with HDL (7). The cholesteryl ester transfer protein (CETP), a hydrophobic glycoprotein transfers cholesteryl esters from HDL to apolipoprotein B (apoB)-containing lipoproteins (8). It seems that the role and importance of CETP in blood is different than other tissues. It has been demonstrated that CETP is related to cell membrane and affects on membrane physical and chemical characteristics by facilitating selective uptake of HDLderived cholesteryl esters (9, 10).

Ranvik *et al* (11) reported that FF has lipid transfer activity, and fractions of FF with this activity stimulate human sperm capacitation and acrosome reaction. It has been shown that this process involves a sterol depletion of the spermatozoa membrane (12). 

The aim of the present study was to investigate the correlation between CETP level in FF and the success of IVF/ICSI techniques in infertile patients undergoing these techniques.

## Materials and methods

A total of 100 patients referred to Tabriz Alzahra Hospital in 2007-2009 were selected for this cross-sectional study. All participants received a screening history, physical examination, measurements of serum hormone levels and blood counts. The subjects' body mass index (BMI) was calculated as measured weight divided by the square of measured height (kg/ m^2^). 

The mean age of subjects was 32±5.46 years with no evidence of any disease. Healthy husband with no smoking habit were defined as including criteria. Uterus abnormalities, positive history of endocrine disease and inflammatory disorders such as thyroid and adrenal disorders, immune system defect and sexual hormones disorders were considered as exclusion criteria in this study. 

Ovarian stimulation was achieved with a GnRH agonist (Sereno, Switzerland) /FSH- long down regulation protocol (13). Controlled ovarian stimulation was started with recombinant human follicle stimulating hormone (rFSH; Sereno, Switzerland) at the third day of menstrual cycle. The daily rFSH dose ranged between 150 and 300 IU, depending on body mass index, age of the women, and the anticipated ovarian response. Dose adjustment was done according to follicular development and serum estradiol levels. Intramuscular hCG (1000IU, Choriomon, Meizler, Brazil) was administered when sonography revealed the average diameter of 3 preovulatory follicle had approached 18- 20mm.

Oocyte retrieval was done 36 h after hCG administration by vaginal ultrasound-guided puncture of the ovarian follicles. The collected oocytes were incubated in 37ºc with 6% CO2 for 3-4 hours and then were used for IVF and ICSI. FF was collected in a sterile tube, was centrifuged at 1500rpm for 5min and kept frozen at -70˚C until analyses.

Oocytes with sporadic cumulus oophorus and zona pellucida and also a clear ooplasm were selected for insemination. The swim-up was used to prepare sperm for IVF (14). Oocytes were inseminated with 250000 sperm per oocytes in the IVF technique (15). In the ICSI group, a single motile sperm were injected into oocyes (16). After 24h, oocyets were separated from surrounding granulosa cells. A maximum of three embryos were transferred at 4-8 cells stages after 48h, under ultrasound guidance. Clinical pregnancy assessed by β-hCG test, 14 days after embryo transfer.

Proportion of the oocytes that had 2 pronuclei was estimated as fertilization rate. This project was approved by the ethics committee of Tabriz University of Medical Sciences. Before the sampling, we took a written consent from studied subjects. 

The levels of follicular HDL-C were measured using enzymatic method by an Abbott Alycon 300 auto analyser. Apolipoprotein A-I concentration determined by immune turbidometric method (Diasys, Germany). The level of CETP was measured by using an Alpco ELISA kit (catalogue number 44-ADPRT-E01). 

**Statistical analysis**

Values are presented as mean± S.D. The one way ANOVA with Tukey post hoc pairwise comparison and Multivariate analysis of variance test were used for comparing means and ratios in different groups. p-values of <0.05 were considered statistically significant. Analysis was carried out using SPSS 11.5 statistical software.

## Results

General characteristics, husband spermogram and FF parameters are shown in table I. The average number of mature oocytes and fertilized oocytes were 8.57±4.19 and 5.0±3.0, respectively. The percentage of pregnancy in the studied population was 24%.

**Figure 1:**
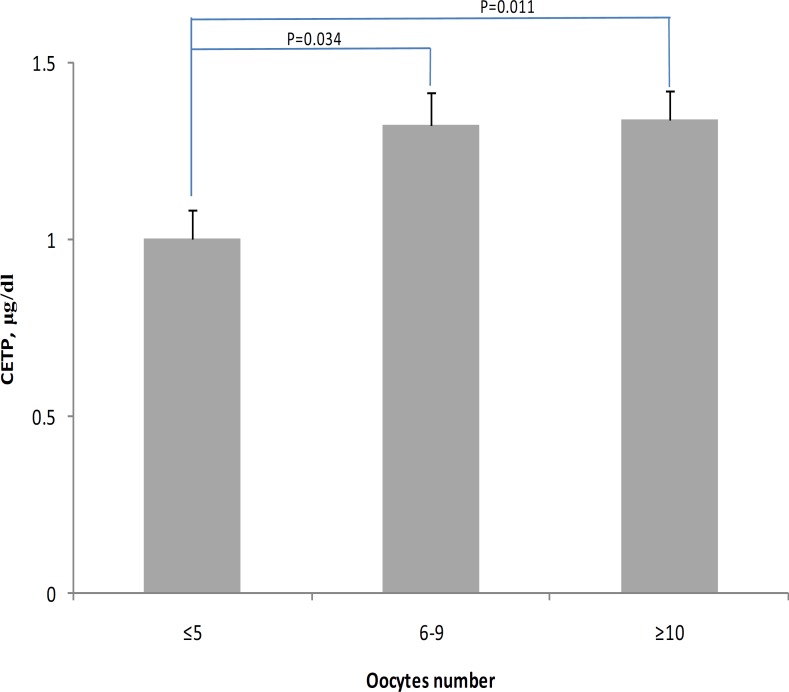
Levels of FF CETP in subgroups with various numbers of mature oocytes. Multivariate analysis of variance with post hoc pairwise comparison. *P*-value adjusted for BMI and age

As shown in table II the age of patients was inversely associated with the number of mature oocytes (r=-0.21, p=0.04) but the level of CETP was positively correlated with the number of mature oocytes (r=0.24, p=0.02). We studied the level of CETP and number of mature oocytes in 3 sub groups with various number of oocytes (group 1, <5; group 2, 6 to 9; and group 3, >10). The level of CETP in group 1 was lower (25%, p=0.01) than group 3 (Figure 1).

There was an inverse significant correlation between BMI and the percentage of fertilized oocytes (r=-0.27, p=0.02). We also found a positive correlation between the percentage of sperm motility and fertilized oocytes (r=0.27, p=0.04). Correlation coefficients of fertilization ratio with sperm count (r=0.23, p=0.05), HDL-C (r=0.21, p=0.06) and CETP (r=0.21, p=0.08) were positive and near to the significant levels (Table III).

**Figure 2:**
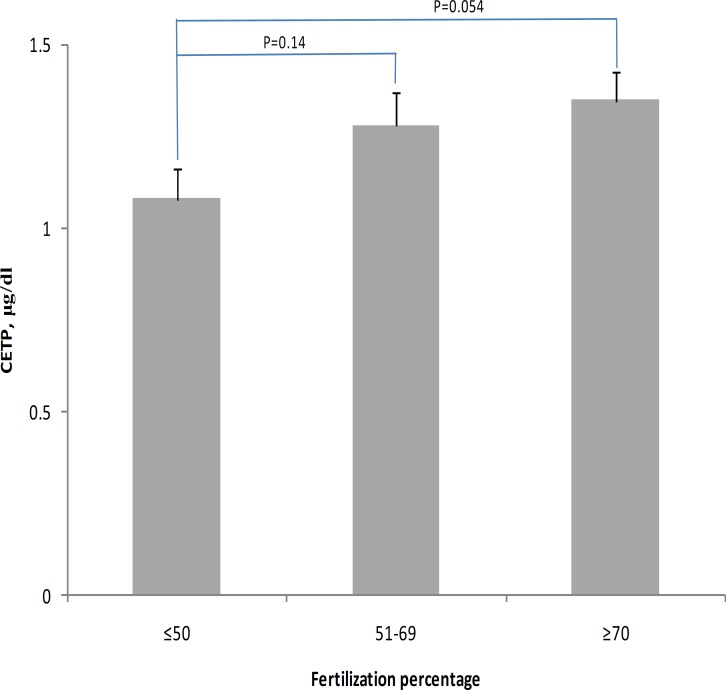
Level of follicular fluid CETP in subgroups with different rate of fertilization. Multivariate analysis of variance with post hoc pairwise comparison. *P*-values adjusted for BMI, Sperm count, Sperm mobility

Our findings also showed that after matching BMI, sperm count, sperm motility and HDL-C, the level of CETP in patients with a percentage of fertilized oocytes less than 50% is relatively lower (18%, p=0.05) than in patients with a percentage of fertilized oocytes> 70% (Figure 2). We found that there were no significant difference between patients with negative and positive pregnancy (p=0.62) in the levels of follicular fluid CETP concentration (Table IV).

**Table I:** Patients' clinical characteristics, biochemical profile of follicular fluid and semen parameters of husbands

**Characteristics**		**Mean ±SD (range)**
**General** Age (years)		31.7±5.46 (21-43)
Body Mass Index (kg/m^2^)		25.5±3.13 (20-34)
**IVF parameters**		
	Mature oocytes	8.57±4.19 (1-19)
	Fertilized oocytes	5.0±3.0 (0-13)
	Biochemical Pregnancy	24%
**Biochemical profile of follicular fluid**		
HDL-C		26±10 (9-24)
Apolipoprotein A-I (mg/dl)		105±19 (14-133)
CETP (µg/dl)		1.23±0.45 (0.1-2.6)
Semen characteristics		
	Sperm count (10^6^/ml)	59.5±30.3 (28-120)
	Sperm motility (%)	66.1±18.4 (10-90)

Values are expressed as mean± SD or percentage (ranges in parenthesis).

**Table II:** Association between parameters and number of mature oocytes

	**p-value**	**r**
Age (years)	0.039	- 0.208
Body Mass Index (kg/m^2^)	0.74	0.034
Apolipoprotein A-I(mg/dl)	0.774	- 0.030
CETP(µg/dl)	0.019	0.240

r, Correlation coefficient

**Table III:** Association between parameters and fertilization rate (FR) in patients undergoing IVF

**Parameter**	**p-value**	**r**
Age (years)	0.436	-0.090
BMI(kg/m^2^)	0.016	- 0.273
Sperm count (10^6^/ml)	0.051	0.235
Sperm motility (%)	0.049	0.266
HDL-C (mg/dl)	0.058	0.217
Apolipoprotein A-I(mg/dl)	0.329	0.113
CETP(µg/dl)	0.076	0.205

FR= (number of fertilized oocyte / number of mature oocytes) ×100 r, Correlation coefficient.

**Table IV:** Level of follicular fluid parameters in clinical positive and negative fertility

	**Non-pregnant** **(n=75)**	**Pregnant** **(n=25)**	**p-value **
Age (years)	31.8±5.6	31.2±5.1	0.63
BMI (kg/m^2^)	25.5±3.2	25.7±3.1	0.74
HDL-C (mg/dl)	26.9±7.0	27.3±6.5	0.78
Apolipoprotein A-I (mg/dl)	103±20	106±15	0.66
CETP (µg/dl)	1.25±0.43	1.19±0.49	0.62

Values are expressed as mean± SD. Biochemical pregnancy was assessed by β-hCG test, 14 days after embryo transfer.

## Discussion

Our study showed that the proportion of the number of fertilized oocytes to the number of mature oocytes and the rate of positive pregnancy were lower than previous studies on population of other countries. 

For example, Mutsubayashi *et al* study showed that the proportion of the number of fertilized oocyte to the number of mature oocytes was 70% (17) and Von Wald *et al* (18) reported a 45% rate of positive pregnancy. Since the success of IVF/ICSI and the occurrence of pregnancy are affected by different environmental factors, these might be attributed to the presence of higher burdens of environmental or genetic risk factors among people of reproductive age in the local population.

There were positive correlations between FF CETP and the number of mature oocytes and percentage of positive fertility. The present study does not clarify the mechanism by which the CETP affect infertility risk status. It can be explained by the effect of CETP on the ability of oocytes to be fertilized and develop into embryos. CETP is a hydrophobic glycoprotein that plays a central role in human HDL metabolism (19). CETP also play an important role in the maintenance of vesicle membrane integrity and/or in membrane fusion events via the transportation of cholesterol from the cytoplasm to the outside of the cell via the cell membrane (20). 

The oocyte membrane has been characterized as important player in the control of sperm-oocyte fusion on the basis of in vitro experiments (21). One possible explanation for the relation of FF CETP with fertilization rate is that CETP promote membrane fusion during in vitro fertilization via the modulating of oocyte membrane function. On the other hand, Ishikawa *et al* found an interesting relationship between cellular mRNA level of CETP and maturation of B cell lymphocytes in germinal centers (22). 

These findings are consistent with the results of the present study showing a positive relation between number of mature oocytes and the level of CETP in FF. It seems that CETP is also involved in follicles and oocyte development via supplying substrate for sexual hormones synthesis in preovulatory ovarian follicles. Accordingly, the importance of lipids metabolism in FF has been identified in previous studies (23, 24).

No statistically significant correlation was seen between clinical parameters and the rate of pregnancy. Considering that the occurrence of pregnancy is affected by factors beyond the characteristics of FF, the absence of a significant relationship between the parameters and the occurrence of pregnancy is probably because of the indirect nature of the relationship and/or the low number of subjects.

According to the results of this study, while there was no association for pregnancy, the amount of FF CETP was correlated positively with the maturity and the percentage of oocyte fertilization. These findings are consistent with the hypothesis that CETP FF plays an important role in cholesterol and lipid metabolism of follicles.
